# Communication skills intervention: promoting effective communication between nurses and mechanically ventilated patients

**DOI:** 10.1186/s12912-017-0268-5

**Published:** 2017-12-15

**Authors:** K. S. Dithole, Gloria Thupayagale-Tshweneagae, Oluwaseyi A. Akpor, Mary M. Moleki

**Affiliations:** 1 School of Nursing, University of Botswana, Gaborone, Botswana; 2 Department of Health Studies, University of South Africa, Pretoria, South Africa; 3 Department of Nursing, College of Medicine and Health Sciences, Afe Babalola University, KM 8.5 Afe Babalola Way, Ado-Ekiti, Ekiti State PMB 5454 Nigeria

**Keywords:** Augmentative and alternative communication, Communication workshops, Intensive care units, Patients, Mechanical ventilation

## Abstract

**Background:**

Patients in the Intensive Care Unit (ICU) often experience communication difficulties - usually associated with mechanical ventilation - resulting in psychological problems such as anxiety, fear, and depression. Good communication between nurses and patients is critical for success from personalised nursing care of each patient. The purpose of this study is to describe nurses’ experience of a communication skills training intervention.

**Methods:**

A convenience sample of twenty intensive care nurses participated in the study. Data was collected by means of interviews with nurses. Data from the interviews were analysed using qualitative thematic content analysis.

**Results:**

Six themes emerged: (1) acceptance of knowledge and skills developed during workshops; (2) management support; (3) appreciation of augmentative and alternative communication (AAC) devices; (4) change in attitudes; and (5) the need to share knowledge with others and (6) inclusion of communication skills workshop training as an integral part of an orientation programme for all nurses.

**Conclusion:**

The findings of this study indicated that the application of augmentative and alternative communication devices and strategies can improve nurse-patient communication in intensive care units. Therefore, the implementation of communication skills training for intensive care nurses should constantly be encouraged and, indeed, introduced as a key element of ICU care training.

## Background

Communication, through verbal and non-verbal means is used to convey messages, share information and establish, strengthen or influence relationships [1]. Non-verbal communication includes, but is not limited to touch, facial expression and tone of voice. Communication forms the basis of a nurse-patient relationship and is an essential element of trust and comfort [1]. Nurses working in intensive care units (ICU) find it especially challenging to communicate with very ill, unconscious, sedated or mechanically ventilated patients [2]. Communication between nurses and these patients requires specific knowledge, skill and commitment.

Despite the fact that nurses spend more time at the patient’s bedside within a multidisciplinary team of health care practitioners (including physicians, speech therapists, physiotherapists, dieticians etc.), traditionally, communication skills training was provided to health care professionals such as the physicians, and did not include nurses [3]. As a result, communication with patients and their families is often neglected and ineffective.

Many studies [4, 5], have alluded to the need for regular training in communication skills for nurses in the ICUs. The same studies have established that patients who have been critically ill, usually remember the nurse patient communication very clearly even if they were unconscious most of the time [6]. This makes it necessary for ICU nurses to have knowledge of the techniques and strategies available to communicate with ICU patients and to apply them in their nursing care.

This study was a result of the findings from research [7] that has shown that communication between nurses and mechanically ventilated patients is minimal [8, 9]. These studies further established that short communication interventions are essential in augmenting communication skills among nurses working with mechanically ventilated patients in the ICU. Based on the recommendations from these studies [8, 9], the researchers found it necessary to develop, implement, and evaluate a workshop-based program on communication skills for nurses, in order to help them attain the skills to communicate effectively with ventilated patients.

This study emerged as a result of the findings from research [7] which revealed minimal communication between nurses and mechanically ventilated patients in ICU, and others [8, 9] that demonstrate that short communication interventions are helpful in enhancing communication skills among nurses working with ventilated patients. Based on the recommendations from these studies [8, 9], it was deemed necessary to develop, implement, and evaluate a workshop-based program on communication skills for nurses, in order to help them attain the skills to communicate effectively with ventilated patients.

A series of six training sessions based on skill assessment of nurses formed the basis of the workshops. The purpose of the training sessions was to promote effective communication between nurses and mechanically ventilated patients, improve patient safety, reduce complaints and lessen work-related stress and frustration.

## Methods

The study was carried out in two ICUs in Botswana from April 2013 to July 2013. A qualitative approach was used. The study was conducted in three phases: phase one was auditing of patients records, procedure manuals and in-service records on communication with ventilated patients. An audit toll was developed from literature on review and communication with colleagues working in the ICU. A quantitative approach was used in Phase 1. Phase 2 used a qualitative approach of semi-structured interviews with 8 nurse managers and 20 nurses working in the two study sites. Phase 3 was development of the intervention which is the focus of this paper.

This paper presents the findings of the third phase, which is the intervention phase and was operationalised in the form of workshops on communication skill training. This phase was informed by the qualitative findings of the second phase, which indicated that nurses working in the ICUs had very limited communication with mechanically ventilated patients. Twenty nurses who participated in qualitative phase two were conveniently selected to participate in the intervention phase three. Nurses attended the workshops (described in detail below) in groups of two or four for one hour daily for six weeks. The lead researcher followed up each participant twice during the six weeks to provide support and any other assistance that was necessary.

### Setting

Two ICUs were chosen from referral hospitals in Botswana, one in Gaborone in the government seat of the Republic of Botswana in the Southern Part of Botswana and one in Francistown, in Northern side of Botswana and in the second largest city. The hospitals were purposively selected, based on the large size of their ICUs, the vast number of patients treated, as well as for geographic diversity. The two ICUs admit patients from all over the country, each admitting an estimated 2095 patients a year, both adults and children. Each ICU has 8 beds, and each has a mortality rate of 3.6%. The majority of patients (1836 per year) admitted in the ICUs are adults [10], with 10% of those admitted to the ICUs being younger than 18 years. All patients admitted in the units are mechanically ventilated. The nurse: patient ratio is 1:2. Two close family members are allowed to visit patients for a one hour visit three times per day.

#### Population and sampling

Convenience sampling technique was used to recruit ten nurses from each hospital. Criteria for inclusion in the study were: (1) nurses who are working in the ICU during the time of data collection, (2) nurses with at least one-year experience of critical care nursing; (3) nurses working at least two consecutive weekday shifts on a regular basis; and (4) willingness to participate in the study. None of the nurses had previous training in augmentative or assistive communication skills.

#### Ethical considerations

The study was approved by institutional boards of the University of South Africa (HSHDC/37/2013), University of Botswana (URB/IRB/1390), Ministry of Health, Botswana (PPME 13/18/1 VOL VII 884) and the two hospitals that participated in the study. The researchers obtained written consent from the participating nurses. Each participant was approached individually and invited to a conference room to explain the purpose of the study, ethical considerations of confidentiality and anonymity, as well as the voluntary nature of their participation in the study.

### Brief summary of the intervention

The interactive intervention workshops were conducted by the first author and they involved role play, return demonstration, group discussion, and exchange of experiences by nurses. The role plays were based on developed scenarios that were adopted and adapted from Rodriguez, Rowe, Koeppel et al. [11]. The scenarios included communication during intubation, suctioning, and tracheotomy care. Emphasis was placed on assessment of the patient for ability to communicate in order for participants to check which of the augmentative and alternative communication (AAC) devices would be appropriate. Workshops were selected as the method of intervention because of findings from other ICU communication studies that demonstrated workshops to be useful tools to empower nurses with the appropriate communication techniques [12]. During the training more than one method was used, including presentation of contact materials, simulation, demonstration and return demonstration, and practice. A lesson plan outlining the intervention is shown in Table 1.

**Table1:** A lesson plan for the communication skills workshops

	Workshop 1		Workshop 2		Workshop 3	
Duration	Session 1	Session 2	Session 1	Session 2	Session 1	Session 2
5 min	Introduction		Introduction		Introduction	
20 min	Role plays on an algorithm, scenario on mechanical ventilation via the endotracheal tube (Annexure P), pen and paper and natural strategies	Sharing experiences	Role plays on scenario for suctioning procedure (Annexure R), alphabet board, word phrase, and natural strategies	Sharing experiences	Role plays on scenario on creating tracheostomy (Annexure T), photo album, picture board and natural strategies	Sharing experiences
10 min	Nurses’ questions and comments on the role play	Nurses practicing on each other	Nurses’ questions and comments on the role play	Nurses practicing on each other	Nurses’ questions and comments on the role play	Nurses practicing on each other
20 min	Return demonstration by nurses		Return demonstration by nurses		Return demonstration by nurses	
5 min	Summary		Summary		Summary	

#### Data analysis

Data analysis was done simultaneously with data collection. Data from the interviews were analysed using qualitative thematic content analysis [13]. Interview text was analysed in several steps, starting with naïve reading of texts. Texts were divided into meaning units and statements that relate to the central meaning and objectives of the study. The meaning units were condensed, abstracted and labelled with codes, which were compared for similarities and differences to develop themes. Quotes were transcribed verbatim, and scripts were given back to some participants to validate the information.

## Results

### Demographic data

Ten nurses from each ICU (*N* = 20), 12 Females and 8 Males. Their age ranged between 25 and 51 years, and their years of experience working in the ICU ranged between 1 and 6 years. Of these nurses 84% held the Diploma in Nursing and 16% held Bachelors of Nursing degree.

#### Themes

Six themes emerged: acceptance of the development of knowledge and skills through workshops; management support; appreciation of the augmentative and alternative communication (AAC) devices; change in attitudes; the need to share knowledge with others and the inclusion of communication skills workshop training as an integral part of an orientation package for all nurses.

### Acceptance of the development of knowledge and skills through workshops

Twenty (100%) nurses indicated the value of workshops. Participants verbalised that they liked the workshop materials, and that the workshops were very educational, particularly on the variety of communication strategies available.

*“We gained a lot from the workshops. We now know the different strategies available to us to help us communicate effectively with mechanically ventilated patients”. This information we will pass it on to our colleagues who did not attend”.*

Twelve nurses indicated that they have started to apply the communication skills they have learned in the workshops. Seven mentioned that they will incorporate communicating with patients as a performance area to be evaluated.

*“I had been unable to come up with objectives for the performance improvement. I am going to include communicating with ventilated patients as my objective”.*

### Management support

Support by managers was identified by the participants as a crucial element of improving nurse-patient communication. Participants verbalised that managers should support them to ensure that materials are available, and that they stay longer in the ICU instead of yearly rotations that are currently the norm.

Eighteen (90%) nurses wished that Management could support them in improving communication with ventilated patients by ensuring that there are AAC devices in the unit such as alphabets and photo albums.

*“Material for AAC devices are good, but we need management to help us by making the supply available... Yes, we shall keep this safe but they would not last long. We need a constant supply…”.*

Ten (50%) of nurses mentioned that as long as they are not deployed to other units they will utilize the AAC devices.

*“The yearly rotations will disturb our focus in using the AAC devices. We have to recommend to the management that they should leave us in one unit for two to three years in order to make the practice of using AAC devices a routine”.*

### Appreciation of the augmentative and alternative communication (AAC) devices

Nurses assured the researchers that they would ensure that the AAC devices used during their workshops, be kept in the unit for every nurse to use when communicating with ventilated patients.

One participant indicated that:

*“We will ensure that these AAC are available in the unit by keeping them safe as long as I am still in this unit”.*

### Change of attitude of nurses towards communication

The nurses reported being excited about communicating with patients and using available tools for communicating. The nurses appreciated the researcher’s initiative for conducting workshops and assured the researchers that they will start using AAC devices and encourage other nurses to communicate with patients.

*“Most of us here are not ICU trained, so if people like you come and empower us, we get motivated. Do not forget that this was just an introduction; keep coming to encourage us to communicate with ventilated patients”.*

### Need to share knowledge gained from the workshops with others

Although 85% (*n* = 17%) of the nurses felt challenged because of time constraints and the type of patients they nurse, they accepted the value of workshops and indicated their intention to share what they learned with other nurses in the unit and nursing students visiting the units.

*“The workshops were very valuable especially to those of us who are not ICU trained. I used to ignore these patients but now I know it is part of my nursing care to communicate with them and convey love and understanding using AAC devices. This knowledge I will share with others”.*

“*Although we are going to try to encourage others to communicate with patients. However, all nurses working in the ICUs need this workshops.”*

Four nurses (20%) mentioned some potential barriers that they might experience after the workshops, they appreciated that caring is not complete without communication.

*“ICU is very challenging environment. The patients we receive in the ICUs are critical and saving life is paramount and hence we forget to communicate. However, after this workshop we will communicate.”*

*“I have realised that giving up while the patient attempted to say something was a mistake. I did not provide full care to my patient.”*

### Inclusion of communication skills workshop training as part of an orientation package for all nurses

The nurses were of the opinion that workshops should be conducted with all ICU nurses and should be included during new nurses’ orientation to the ICU.

*“During our orientation, emphasis on communication was not done. I believe they took for granted that all nurses can communicate with ventilated patients without difficulties”.*

Eighteen (90%) nurses felt that communication is vital in nursing, and that they have realized that when they do not communicate with the ventilated patients, there is a deficit in the care provided. They felt that every new nurse should be orientated on nurse-patient communication and attend communication skill workshop.

One participant had this to say:

*I carried out procedures silently or ignored patient when I could not understand them. Now I appreciate that my care was incomplete. I strongly believe that emphasis on communicating with ventilated patients should be extended to new nurses.*

## Discussion

The majority of the participants expressed the value of the workshops conducted. The nurses indicated that they feel inclined to continue practicing what they have learned in the workshops. This addresses a need demonstrated in a number of studies on nurse’s perceptions of nurse-patient interactions that show that nurses perceive communication with intubated patients as a waste of time and time consuming [14, 15].

The findings of this study are similar to the findings of a study conducted in the USA on nurses’ perceptions of communication training in the ICU [16]. The US study reported poor attitude, unavailability of skill training workshops, and a lack of communication materials as deterrents to communication, which was echoed in the findings of this Botswana ICU study. The one difference observed between the two studies was that, in the Botswana ICU study, values changed as participants’ verbalised valuing communication with sedated patients. This was not observed in the previous study.

The nurses felt empowered to assist patients by reducing anxiety and stress through better communication. This change in attitude is in line with the study of Happ, Garrett & Tate et al. [9] that used communication skills training to improve nurse-patient interactions. Nurses are expected to provide quality care in intensive care as well as to empower their patients. This is possible if their communication is supported with the use of AAC devices which have been found to be effective in assisting patients who had to be admitted to the intensive care unit. One positive attribute important in communicating with ventilated patient is patience; even when using simple low technological devices such as a communication board [17]. Although some nurses may have a negative attitude towards nurse-patient communication in the ICU, encouragement and motivation from other nurses and nurse managers can improve the situation.

The nurses who attended the training demonstrated enthusiasm by verbalising that such training should be continuous as supported by Happ et al. [5]. It is evident in this study that effective communication by nurses can be influenced by many factors such as the level of education and psychological conditions in which the nurse work [18]. Many studies emphasise the role of encouragement in nurturing creativity, innovation and opportunity for lifelong learning in the workspace, such as the ICU [19, 20]. It is in this regard that continuous training for all nurses on communication skills should be regular and mandatory for ICU nurses. A qualitative study by [21] emphasised the importance of teaching nurses, including nursing students, about communication skills such as active listening in the ICU. In support of this study, Happ [5] advocates that ICU nurses should be educated on nurse-patient communication. These studies support the findings of this study, that to train all nurses working in the ICU on communication skills is essential.

## Conclusion

The participants valued communication workshops and it was apparent that if they could be supported through in-service education and by ensuring that there are available communication materials in the unit for effective nurse-patient communication. There is a need for Management to provide ICU nurses with at least low technological AAC devices in order to facilitate effective communication with ventilated patients.

### Limitation of the study

The study was conducted in two referral hospitals in Botswana which covers a large geographical area and admits patients across the country. However, nurses working in smaller ICUs with fewer patients may have yielded different results. The use of a smaller sample and reliance in interviews also served as a limitation as the findings may not be generalised. The researchers appreciate that the participants were interviewed before completion of the intervention. It might have been too early to determine the sustained change in nurses’ attitudes towards the effort to communicate effectively with ventilated patients.

### Recommendations

The findings of this study prompted the following recommendation:

1. There is a need for a follow up study after the intervention to assess nurses’ perceptions and experiences regarding nurse-patient communication.
2. A follow up study is needed to determine whether the nurses’ competencies after communication skill training improved.
3. The AAC devices must be made available in order to assist nurses to communicate with less difficulty with ventilated patients in the ICU.
4. There is a need to incorporate useful communication methods and strategies in the nursing curriculum and in-service education programmes.

### Implications for nursing practice, nurse education and healthcare policy

The study findings have implications for policy development and training of ICU nurses on effective communication skills with ventilated patients. Continuing education on nurse-patient communication is a necessity for enhancing good communication and as well as improved quality of patient care. It is of utmost importance that guidelines on nurse-patient communication should be established in ICUs. The study provided evidence for the need to ensure continuous education and training of nurses in communication skills. The nurses in this study were motivated and willing to continue with what they learned during communication workshops, thus, a follow up study is needed to find out if the competencies in communication skills improved after the workshops with the nurses.

## Abbreviations

AAC: Augmentative and alternative communication
ICU: Intensive care unit

## References

[1] Kourkouta L, Papathanasiou IV (2014). Communication in nursing practice. Mater Sociomed.

[2] Ayuso-Murillo D, Colomer-Sanchez A, Herrera-Peco I (2017). Communication skills in ICU and adult hospitalisation unit of nursing staff. Enferm Intensiva.

[3] Boyle DA, Anderson WG (2015). Enhancing the communication skills of critical care nurses:focus on prognosis and goals of care discussions. Clin Commun.

[4] Bramhall E (2014). Effective communication skills in nursing practice. Nurs Stand.

[5] Happ MB, Garrett K, Sereika S (2012). Nurse-patient communication interactions in the intensive care unit. Am J Crit Care.

[6] Gauntlett R, Laws D (2008). Communication skills in critical care. Contin Educ Anaesth, Crit Care Pain.

[7] Dithole KS, Sibanda S, Moleki MM, Thupayagale-Tshweneagae G (2016). Nurses’ communication with patients who are mechanically ventilated in intensive care: the Botswana experience. Inter Nurs Rev.

[8] Boscart VM (2009). A communication intervention for nursing staff in chronic care. J Adv Nurs.

[9] Happ MB, Garrett KL, Tate JA (2014). Effect of a multi-level intervention on nurse-patient communication in the intensive care unit: results of the SPEACS trial. Heart Lung.

[10] Statistics Botswana. Statistics Botswana annual report 2013/2014; 2014.

[11] Rodriguez CS, Rowe M, Koepal B, Thomas L, Troche MS. Development of a communication intervention to assist hospitalisezed suddenly speechless patients. Technol Health Care. 2012:489–500. 10.3233/THC-2012-0695 PMC4018578 23187014

[12] Slatore CG, Hansen L, GanziniL PN, Osborne ML, Chesnutt MS, Mularski RA (2012). Communication by nurses in the intensive care unit: qualitative analysis of domains of patient-centered care. Am J Crit Care.

[13] Bernard HR. Research methods in anthropology: qualitative and quantitative approaches. Walnut Creek: AltaMira Press; 1994.

[14] Leathart AJ. Communication and socialisation:and exploratory study and explanation for nurse-patient communication in an ITU. Intensive Crit Care Nurs. 1994:93–104. 10.1016/0964-3397(94)90004-3 8012157

[15] Magnus VS, Turkington L (2006). Corrigendum to communication interaction in ICU—patient and staff experiences and perceptions. Intensive Crit Care Nurs.

[16] Radtke JV, Tate AJ, Happ MB (2012). Nurses perceptions of communication training in the ICU. Intensive Crit Care Nurs.

[17] Patak L, Gawlinski A, Fung NI, Doering L, Berg J, Henneman EA (2006). Communication boards in critical care: patients’ views. Appl Nurs Res.

[18] Sabiston JA, Laschinger HK (1995). Staff nurse empowerment and perceived autonomy:testing Kanters theory of structural power in organizations. J Nurs Adm.

[19] Cowden T, Cummings G, Profetto-McGrath J (2011). Leadership practices and staff nurses’ intent to stay: a systematic review. J Nurs Manag.

[20] Isfahani SS, Hosseini MA, Khshknab MF, Peyrovi H, Khanke HR. Nurses creativity: advantage or disadvantage. Iran Red Crescent Medical Journal. Online. 2015. 10.5812/ircmj.20895 PMC4353217 25793116

[21] Barrere C (2007). Discourse analysis of nurse-patient communication in a hospital setting. J Nurses Staff Dev.

